# Estimation of Tree Size Diversity Using Object Oriented Texture Analysis and Aster Imagery

**DOI:** 10.3390/s8084709

**Published:** 2008-08-11

**Authors:** Ibrahim Ozdemir, David A. Norton, Ulas Yunus Ozkan, Ahmet Mert, Ozdemir Senturk

**Affiliations:** 1 Suleyman Demirel University, Faculty of Forestry, 32269, Isparta, Turkey; 2 School of Forestry, University of Canterbury, Private Bag 4800, Christchurch 8140, New Zealand; 3 Istanbul University, Faculty of Forestry, Bahcekoy, Istanbul, Turkey; 4 Suleyman Demirel University, Remote Sensing Research and Application Centre, Isparta, Turkey E-Mails: david.norton@canterbury.ac.nz (D.A.N); ulasyunus@yahoo.com (U.Y.O.); amert@sdu.edu.tr (A.M.); ozdemirsenturk@yahoo.com (O.S.)

**Keywords:** Tree size diversity, remote sensing, brutian pine, texture analysis, image segmentation

## Abstract

This study investigates the potential of object-based texture parameters extracted from 15m spatial resolution ASTER imagery for estimating tree size diversity in a Mediterranean forested landscape in Turkey. Tree size diversity based on tree basal area was determined using the Shannon index and Gini Coefficient at the sampling plot level. Image texture parameters were calculated based on the grey level co-occurrence matrix (GLCM) for various image segmentation levels. Analyses of relationships between tree size diversity and texture parameters found that relationships between the Gini Coefficient and the GLCM values were the most statistically significant, with the highest correlation (r=0.69) being with GLCM Homogeneity values. In contrast, Shannon Index values were weakly correlated with image derived texture parameters. The results suggest that 15m resolution Aster imagery has considerable potential in estimating tree size diversity based on the Gini Coefficient for heterogeneous Mediterranean forests.

## 1. Introduction

Biodiversity conservation has become an increasingly important issue in forest management, with forest managers now having to include biodiversity considerations within existing management plans [1, 2]. Stand diversity, especially variation in tree height and diameter, is an important consideration in biodiversity conservation in forested landscapes [3, 4]. A large diversity in tree sizes can provide a wide range of habitat for wildlife and continuously supplies dead trees which are vital for ecosystem processes such as nutrient cycling. Furthermore, forests with greater tree size diversity usually have greater aesthetic and recreational values [5].

Differences in tree size diversity may be due to a variety of factors including species composition, age differences and disturbance history. Management activities and the effects of insect and fungal pathogens may also affect tree size diversity. Wood oriented management systems have a homogenizing effect on stand structures as the goal is to grow even-aged stands. Furthermore shrubs and deciduous trees are removed by thinning to favor conifer trees, which are economically more valuable. Consequently, wood oriented silvicultural treatments have decreased the variability of size and tree species distributions in comparison with unmanaged stands [6].

Diameter at breast height (DBH), tree height, and crown depth and width can all be used to describe tree size [7]. DBH is widely used as it is straightforward to measure and highly correlated to the other parameters [8, 9]. A variety of indices including Shannon's index, Simpson index, Gini coefficient, Margalef index, McIntosh index, Berger-Parker index, Shannon evenness, McIntosh evenness and coefficient of variation have been used to quantify tree size diversity in previous studies (7; 10-12]. These indices can be used in various forestry applications including; i) comparison of habitat quality for wildlife in different stands, ii) monitoring of changes in tree size diversity over time, iii) determining the impact of different silvicultural treatments on stand structure, and iv) defining the appropriate silvicultural treatments for different stands [7].

Mapping and monitoring tree size diversity over large areas is crucial to forestry. The use of ground-based methods to determine and map tree size diversity for all stands in a landscape is, however, both expensive and time consuming. Satellite remote sensing technologies provide a powerful alternative for providing such information. Satellite image data are a cost efficient source of information especially for large-area forest inventories, and have been widely applied in forest mapping and monitoring [13-22]. The value of remote sensing as an efficient tool in mapping biodiversity at the habitat scale also has been emphasized in several studies [e.g., 23, 24]. More recently, research has focused on the potential of using remote sensing images for assessment of biodiversity at the plot level. For example, Bawa et al. (2002) reported that there is a statistically significant relation between the species diversity and the Normalized Difference Vegetation Index (NDVI) of IRS 1C imagery (r=0.66, p<0.01) and NDVI may be used to characterize areas of high and low species richness of trees in tropical forests where biodiversity losses are high [25]. Similarly, Levin et al. (2007) showed that there are significant correlations between plant species richness and the NDVI of Aster data (rs=0.89, p<0.01) and the NDVI of Landsat ETM+ data (rs=0.93, p<0.01) in a mountainous region in Israel [26].

Object oriented image analysis is a widely used tool for mapping and monitoring forests [27-29], and for estimating a range of forest stand attributes [30]. The use of image segmentation algorithms offered by Definiens Professional software represents a powerful tool for extracting spectral, spatial and textural features at object level. These variables can be used for estimating stand structure parameters in a cost-efficient manner. In this paper we hypothesize that greater tree size diversity occurs in stands that form more textured image objects which will allow modeling of tree diameter diversity indices using textural features derived from satellite imagery. Specifically we investigate the relationships between the textural features derived from Aster imagery and indices characterizing tree size diversity obtained from ground-based sample plots in a typical Mediterranean forested landscape in Turkey.

## 2. Materials and Methods

### 2.1. Study Area

The study area (centered on 37°18′50″N, 30°44′50″E, 350 – 1200 m a.s.l.) is mostly composed pure *Pinus brutia* stands. *Cedrus libani* A. Rich., *Abies cilicica* (Ant. et Klotsch.) Carr., *Juniperus excelsa* M. Bieb. and some oak species (including *Quercus coccifera* L. and *Quercus cerris* L.) also form stands in the study region. Other natural tree species include *Alnus glutinosa* subsp. *antitaurica* Yalt., *Platanus orientalis* L., *Liquidambar orientalis* Mill. and *Salix alba* L., which are mostly located in riparian zones. The *Pinus brutia* stands have been intensively managed for approximately 40 years and are mostly structurally simple. In contrast the stands of *Cedrus libani*, *Abies cilicica*, *Juniperus excelsa* and *Quercus cerris* are more natural in composition and are structurally diverse. Thus, the study region covers both managed and unmanaged forest stands and represents a wide range of tree size diversity.

### 2.2. Creating of segmented images

The analyses were based on the three bands with 15 m spatial resolution of Aster (Advanced Spaceborne Thermal Emission and Reflection Radiometer) satellite data with an image acquisition date of May 5th 2007. The spectral ranges of the three bands were 0.52-0.60 μm (green), 0.63-0.69 μm (red) and 0.76-0.86 μm (near infrared). Image pre-processing including atmospheric correction and orthorectification were applied by the supplier in order to correct distortions and degradations resulting from the image acquisition process and no further image pre-processing was undertaken.

The texture features were extracted from the image segments generated by the multi-resolution segmentation approach developed by Definiens image processing software. Segmentation is an algorithm which creates meaningful objects in an image by grouping the individual pixels according to their spatial and spectral properties and is performed using the scale parameter and homogeneity criteria. The scale parameter is an abstract term which determines the maximum allowed heterogeneity of the resulting image objects. While the scale parameter is increased, the homogeneity of segments decreases and the standard deviation within the resulting image objects increase [31].

The homogeneity criterions are the color, shape, compactness and smoothness in Definiens. It is recommended that the color criterion should be used as much as possible while keeping the shape criterion as high as necessary to produce image objects that suit the purpose. In other words, the color criterion is the most important for forming meaningful objects because the spectral information is the primary information contained in an image [32]. Nevertheless, the amount of weight color and shape information should be given is difficult to define in this algorithm. Therefore, choosing optimal algorithm-associated parameters towards high-quality segmentation for a given feature type is an essential step [33]. In order to determine the best composition of homogeneity criterions that is suitable for this study and the image data used, the different options of homogeneity criterions were visually evaluated, with nine color and shape combinations tested multifariously (0.1 color – 0.9 shape; 0.2 color – 0.8 shape; 0.3 color – 0.7 shape; 0.4 color – 0.6 shape; 0.5 color – 0.5 shape; 0.6 color – 0.4 shape; 0.7 color – 0.3 shape; 0.8 color – 0.2 shape; 0.9 color – 0.1 shape) in conjunction with three different compactness and smoothness combinations (0.3 compactness – 0.7 smoothness; 0.5 compactness – 0.5 smoothness; 0.7 compactness – 03 smoothness). Landscape patterns such as the stand borders in the forest maps for the study area were also taken into consideration in this assessment. As a result of the visual evaluation, 0.8 color – 0.2 shape and 0.5 compactness – 0.5 smoothness were selected as providing the best homogeneity combination to create meaningful objects.

The segmentations at eleven different scales were conducted by modifying the scale parameter in order to determine the optimal segment scale that allows the best modeling of tree size diversity. The eleven scale parameters tested in this study were 10, 15, 20, 25, 30, 35, 40, 45, 50, 55, and 60. We accepted this range based on the existing forest map. The stand borders in the forest map were visually compared with the resulting segments after each segmentation process. We did not repeat the segmentation process because we understood that the segments created using the scale parameters which are less than 10 and more than 60 did not form meaningful objects; segment scales >60 delineated objects capturing both fruit orchards and forests while segment scales <10 generated objects that were too small to match image features with the ground sampling plot data.

### 2.3. The diversity indicates

A large number of diversity indices can be used to characterize tree size diversity within a stand [7, 10-12]. We used Shannon index and Gini coefficient in this study because they have been widely used in previous studies. These diversity indices were calculated based on tree diameter at breast height (1.3 m – DBH) within forest sampling plots.

The Shannon index [34] is a widely used in ecological studies as a measure of tree size diversity (7, 10, 11]. The proportion of basal area per diameter classes is used in this index. This index depends on the selected size class width [35]. Shannon index value decreases when the number of classes decreases with increasing class width [10]. However, there is no agreement in the literature concerning what class width should be used. For example, Lexerod and Eid (2006) used 2 cm, Varga et al. (2005) used 4 cm, and Wikstrom and Eriksson (2000) used 5 cm diameter class widths [4, 7, 10]. In this study, we used 4 cm class widths to characterize basal area distribution. The maximum value of the Shannon is ln (N) providing that basal area is evenly distributed over all diameter classes and the minimum value is zero when all trees are in only one diameter class. The Shannon index (H′) is calculated as:

(1) $$H^{\prime }=-\sum_{i=1}^{N}p_{i}\;\ln(p_{i})$$

where pi is proportion of basal area in size class i; and N is number of diameter classes

Since the sensitivity of the Shannon index to the change in class width is uncertain, we also used the Gini coefficient as it does not require arbitrary classified diameter classes and has been proposed for calculating tree size diversity [7]. The Gini coefficient was originally developed in economics for determining inequality of income distribution and has been widely used in the measurement of heterogeneity in tree sizes [7, 11]. The minimum value of this coefficient is zero when all trees have equal size, while the theoretical maximum value is 1 when all trees except one have a value of zero (extreme inequality). Lexerod and Eid (2006) suggest use of the Gini coefficient in forest management; comparing tree size diversity in different stand, evaluating changes in tree size diversity over time, and determining the impact of different silvicultural interventions on tree size diversity [7]. The Gini coefficient (GC) is calculated as:

(2) $$GC=\frac{\overset{n}{\underset{j=1}{\text{∑}}}(2j-n-1)ba_{j}}{\overset{n}{\underset{j=1}{\text{∑}}}ba_{j}\;(n-1)}$$

where, baj is basal area for tree in rank j (m2 ha-1); and n is total number of trees; and j is the rank of a tree in order from 1,…,n.

Diversity indices were determined for 541 geo-referenced circular sampling plots that had been measured as part of forest management plan development by forest inventory teams in 2007. In the Turkish forest inventory system, the sampling plots are systematically distributed at 300 m intervals over the forested areas of a planning unit with plot sizes of 0.04 ha, 0.06 ha and 0.08 ha depending on the crown closure degrees of stands. The sampling plots as point data were overlaid with the eleven segmented images using GIS. A 20 m buffer zone was created from segment boundaries in order to reduce the spatial error resulting from GPS measurement. The texture information derived from a segment was then matched with the diversity indices of sampling plots which are situated in that segment. If a segment has more than one sampling plot, the arithmetic mean of these plots was used in the correlation analysis.

### 2.4. The texture variables

The textural properties of the segments from the eleven segmented images were determined based on the approach of “Texture after Haralick”. The texture parameters were calculated for all pixels of an image object based on the grey level co-occurrence matrix (GLCM) that is a tabulation of how often different combinations of pixel grey levels occur, in a given direction, in an image object. The grey-level co-occurrence matrix can reveal certain attributes pertaining to the spatial distribution of the grey levels in an image object. Several statistical measures can be also derived from the GLCM. Hall-Beyer (2007) separated these into three main groups; i) contrast, ii) orderliness and iii) descriptive statistics [36]. The GLCM parameters including homogeneity, contrast and dissimilarity belonging to the contrast group use weights related to the distance from the GLCM diagonal. Contrast increases when the elements of a matrix are away from the diagonal, meaning greater differences between the grey levels of pixels in an image object. Therefore, the contrast group measures were examined in this work because they are considered to be of high potential for predicting tree size diversity.

Three GLCM contrast parameters were calculated for each band (0.52-0.60 μm, 0.63-0.69 μm and 0.76-0.86 μm) separately. Since a single GLCM using one direction might not be enough to describe the textural features of an image object, the three GLMC operations were performed based on the four directions as horizontally (90°), vertically (0°), and two diagonally (45° and 135°) [31].

Every GLCM is normalized according to the formula below [36];

(3) $$P_{i,j}=\frac{V_{i,j}}{\overset{N-1}{\underset{i,j=0}{\text{∑}}}V_{i,j}}$$

Where; i: the row number; j: the column number; Vij; the value in the cell i,j of the matrix; Pij: the normalized value in the cell i,j; N: the number of rows or columns.

The GLCM homogeneity measures the closeness of the distribution of elements in the GLCM to the GLCM diagonal. The GLCM homogeneity of an image object is high if GLCM concentrates along the diagonal. It decreases exponentially according to their distance to the diagonal. The formula of GLCM homogeneity is [36];

(4) $$\sum_{i,j=0}^{N-1}\frac{P_{i,j}}{1+{\left(i-j\right)}^{2}}$$

GLMC Contrast is a measure of the amount of local variations in the grey-level co-occurrence matrix. It is the opposite of the GLMC Homogeneity and increases exponentially when i-j increases. The formula of GLMC Contrast is [36];

(5) $$\sum_{i,j=0}^{N-1}P_{i,j}\;{\left(i-j\right)}^{2}$$

The Dissimilarity is similar to GLMC Contrast, however increases linearly as i-j increases. It is high if the local region has a high contrast. The formula of GLMC Dissimilarity is [36];

(6) $$\sum_{i,j=0}^{N-1}P_{i,j}\left|i-j\right|$$

### 2.5. Statistical analysis

Correlation analysis was performed to determine if there was a significant relationship between the texture parameters and the diversity indices. Pearson's product moment correlation coefficient (r) was used to assess the relationship between the variables. A two-tailed p value was used to calculate statistical significance; a value of P<0.05 was taken to be significant. Therefore, the variables that show the highest correlation were identified to establish a regression model for mapping tree size diversity. The Gini coefficient was used as the dependent variable while the texture parameter was used as an explanatory variable for the regression model. Since the linear regression analysis assumes all variables have a normal distribution, the Kolmogorov-Simirnof Z test was applied to the variables. If P>0.05, a variable was accepted as having a normal distribution. The significance of the slope of the regression line was determined by the t-statistic.

## 3. Results

An initial examination of the relationship between Gini coefficient and Shannon index values for the sample plots showed this to be non-linear. Therefore a Spearman's correlation test was used to determine the strength of the relationships between these two variables, with a correlation to rs = 0.55 (P<0.01).

**Figure 1.**
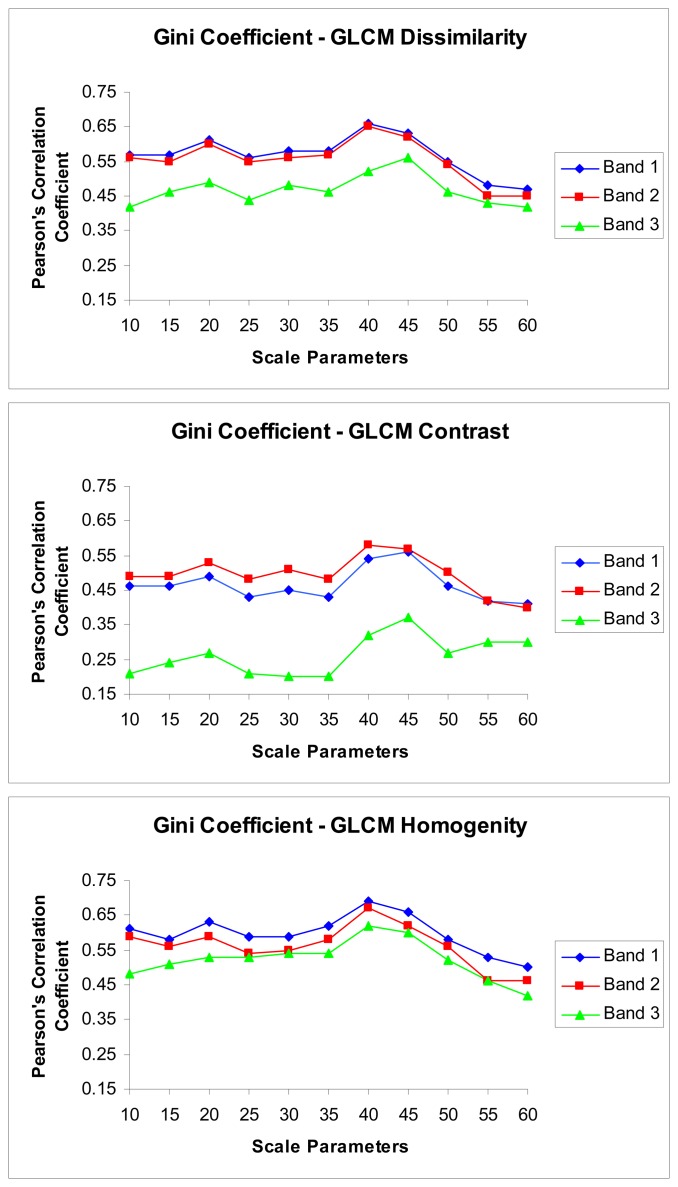
The Pearson's correlation coefficients of the relations between the Gini coefficient and the texture parameters for the eleven segment level with regard to the bands.

Statistically significant linear relationships occurred between the textural parameters and the diversity indices, with the Gini coefficient more strongly correlated (highest r = 0.69, P < 0.01) than the Shannon index (highest r = 0.35, P < 0.01) with image derived texture parameters, and for this reason the rest of this paper focuses on the Gini coefficient. For the Gini coefficient, the strongest correlations were with a scale parameter of 40 (Fig. 1). For this scale parameter, the correlation between Gini values and the texture parameters was highest for GLCM Homogeneity (r = 0.69, P < 0.01), intermediate for GLCM Dissimilarity (r = 0.65, P < 0.01) and lowest for GLCM Contrast (r = 0.58, P < 0.01).

The texture parameters of the GLCM Homogeneity and the GLCM Dissimilarity derived from the green band (0.52-0.60 μm) were most strongly correlated with the Gini coefficient, while for GLCM Contrast derived from the red band (0.63-0.69 μm) were most strongly correlated with the Gini coefficient (Fig. 1). However, the Near Infrared (NIR) band (0.76-0.86 μm) yielded the lowest r values for all texture parameters. This was especially apparent for GLCM Contrast, with markedly lower correlation r values than in any of the others (Fig. 1). The other interesting result is that the r values of the three bands obtained using GLCM homogeneity were closer to each other across all scales when compared with the GLCM Dissimilarity and the GLCM Contrast results (Fig. 1).

**Figure 2.**
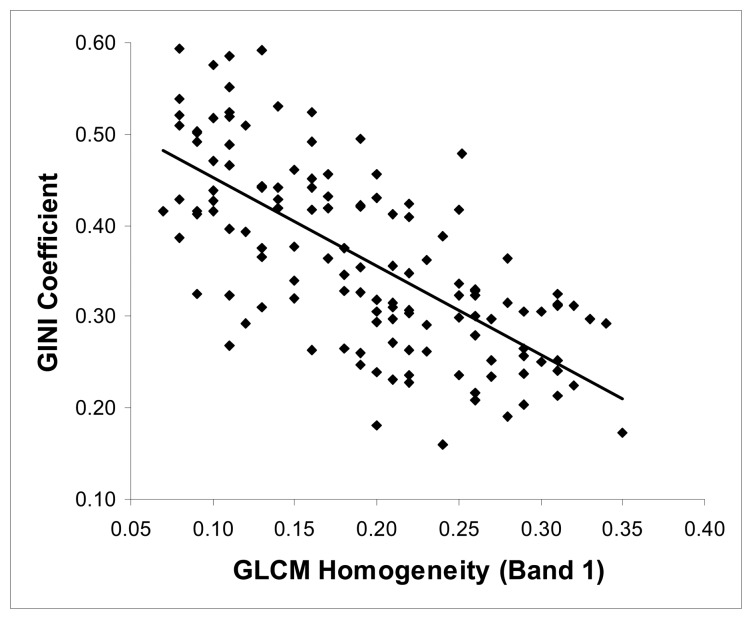
The scatter plot of Gini Coefficient values against the corresponding GLCM Homogeneity values of band 1.

As a result, the GLCM Homogeneity values of band 1 (0.52-0.60) derived from the segments generated by scale parameter of 40 were chosen as the explanatory variable for predicting the Gini coefficient because the highest r value were obtained from this relation. Therefore, a regression analysis was performed to determine the ability of GLCM Homogeneity to predict Gini coefficient values and be used as a tool for mapping tree size diversity. Least squares linear regression analysis was used because visual examination of the scatter plot suggested that relations between the GLCM Homogeneity and the Gini coefficient were linear (Fig. 2). According to the Kolmogorov-Simirnof Z test, both the Gini coefficient and the GLCM Homogeneity values were found to be normally distributed (P > 0.05). The resultant regression was significant, with a negative slope, indicating that image texture values decrease as Gini coefficient increases and that GLCM Homogeneity values of band 1 can be used to predict Gini values.

The regression equation is; *Gini Coefficient* = -*0.9719* × *GLCM Homogeneity* +*0.5494*

**Figure 3.**
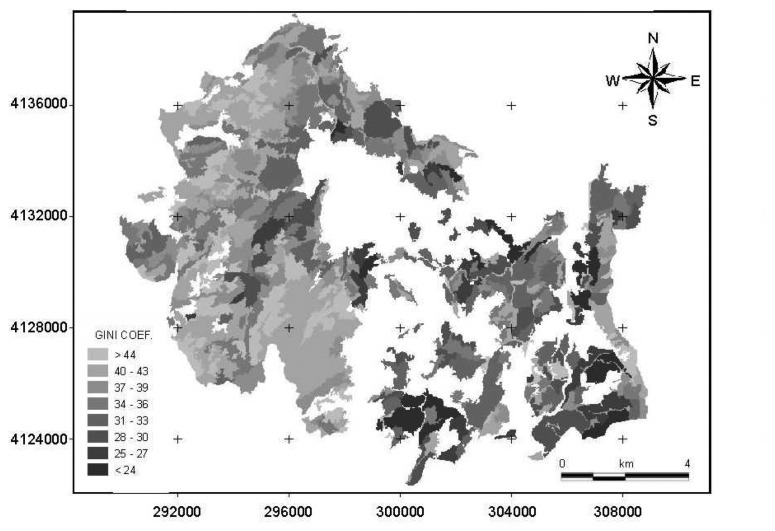
A tree size diversity map based on the Gini coefficient values estimated from GLCM Homogeneity values of the image segments of band 1 generated from scale parameter of 40.

This regression equation was then used to produce a spatial map of tree size diversity (Fig. 3) based on Gini coefficient estimated from GLCM Homogeneity.

## 4. Discussion and Conclusion

### 4.1. Comparison of Gini coefficient and Shannon index

Lexerod and Eid (2006) observed a stronger correlation between the Shannon index and Gini coefficient than observed here (rs = 0.73 cf. 0.55). This difference may result from differences in the tree species and forest conditions of the two study regions. For example, the typical Mediterranean forests we studied have degraded stands because of long-standing anthropogenic impacts, with crown closure of less than 40 %. In the area studied here, the average tree density in degraded stands is 150 individuals ha-1 which results in a small number of trees inside a sampling plot. Thus, Shannon index values calculated from these sampling points may be influenced by sample size thus weakening the relation between Gini coefficient and Shannon index. The other reason may be differences in size classes used. Lexerod and Eid (2006) used 2 cm diameter class width while 4 cm was used here, which might also influence the association between the Gini coefficient and Shannon index [7].

The most appropriate diameter class width to use for calculating the Shannon index is still under debate [10]. It is clear that when the class width increases, the number of classes become too low for the index to be meaningful. Conversely, when the size class width is very small (e.g., 1 cm) the index may lose its sensitiveness to tree size diversity. This is clearly illustrated by comparing three example plots representing low, moderate and high tree size diversity (Table 1). The Gini coefficient is sensitive to the variation of tree size diversity, while the Shannon index yields incongruous values which are strongly influenced by diameter class width (Table 1). This is only a simple example using only three plots and a more comprehensive investigation is required to better determine the effect of class width on the Shannon index. Such an evaluation should also consider the effect of sample size.

The greater utility of Gini coefficient in determining tree size diversity based on basal area measurements in comparison to other indices such as Shannon index, Simpson, Margalef, McIntosh, Berger-Parker, Shannon evenness and McIntosh evenness has been emphasized in other studies [e.g., 7]. Consequently, it can be concluded that the Gini coefficient which does not require an arbitrary classified diameter classes is a more reliable parameter for quantifying tree size diversity. Furthermore, existing sample plot data measured during timber management plan inventories can be used in the calculation of the Gini coefficient because it this coefficient is less sensitive to the number of trees in a sampling plot.

### 4.2. The relations between the texture parameters and Gini coefficient

**Figure 4.**
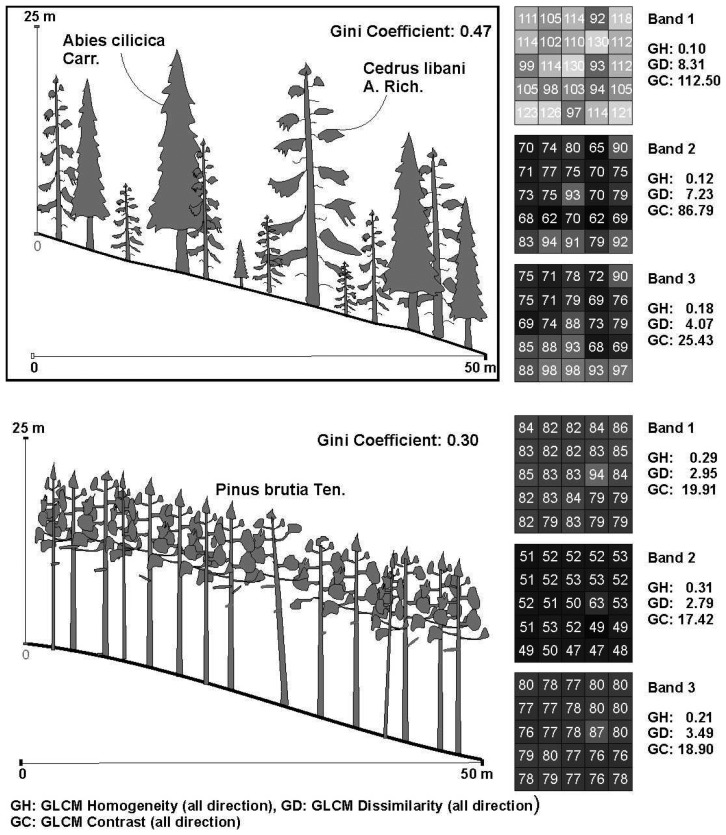
The spectral responses and Gini coefficient of the typical both managed and unmanaged stands and their corresponding texture values with regard to the bands.

A remarkable result from this study is that the relation between the Gini coefficient and the GLCM Homogeneity and Dissimilarity values of the green band (0.52-0.60) is stronger than the other bands, while the texture parameters of the NIR band (0.76-0.86 μm) are the least strongly correlated. Surprisingly, the NIR band yields extreme GLCM Contrast texture values in the segments which are adjacent to the border of study area. The superiority of the green band might arise from its sensitiveness to the bare soil and exposed rocks. Therefore, it can be concluded that the unmanaged stands with high tree size diversity and less crown closure yield a more textured image in the green part of electromagnetic spectrum due to its distinction capability. In visually inspection on the image, it is easily noticed that the NIR band in some segments exhibits uncongenial texture values compared with bands 1 and 2. The two image segments belonging to both an unmanaged mixed stand consisted of *Cedrus libani* and *Abies cilicica*, and a managed *Pinus brutia* stand illustrate their spectral responses to these bands (Fig. 4). In this example, the texture features of band 1 and band 2 is very definite as is expected. However, in the band 3, although they have a different structure, the texture values belonging to the two forests are very similar to each other. Consequently, this makes the development of a statistically robust regression model using the texture values of the NIR band to predict the Gini coefficient difficult, particularly when CLCM Contrast and GLCM Dissimilarity values are used.

The results presented here indicate that Aster imagery with 15 m resolution is promising for estimating the Gini coefficient as an indicator of tree size diversity for heterogeneous Mediterranean landscapes. The highest correlation coefficient (r=0.69) was found between the GLCM Homogeneity of band 1 and the Gini coefficient using the segments generated by the scale parameter of 40. This relation can allow mapping tree size diversity based on Gini coefficient over large geographical areas. Such satellite derived maps can then be used to evaluate changes in tree size diversity over time and to determine the impact of different management interventions on tree size diversity at the landscape scale. We are not aware of any other research that has investigated the relationship between tree size diversity and remote sensing imagery. Therefore, this study suggests another and potentially very important application of remote sensing in forest management and conservation.

**Figure 5.**
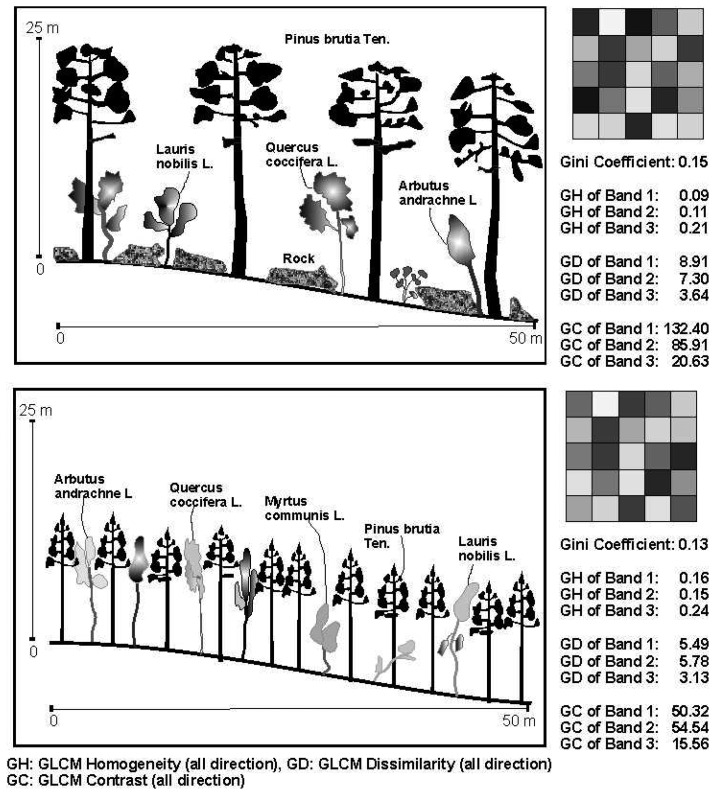
Two example stands with low tree size diversity, however, some types of maquis vegetation enter into the brutian stands because they reach enough light. Therefore, they creates more textured image due to their varying spectral characteristics.

When the extreme values in the scatter plots of GLCM Homogeneity of band 1 against Gini coefficient are inspected, two stand types are evident as outliers. These are; i) mature *Pinus brutia* stands with low crown closure located on rocky surfaces and ii) young *Pinus brutia* stands that have not yet attained crown closure. In this latter case, maquis vegetation including *Myrtus communis* L., *Arbutus andrachne* L., *Erica arborea* L., *Quercus coccifera*, *Ceratonia siliqua* L. and *Lauris nobilis* L. grow within the young *Pinus brutia* stand. Although the calculated Gini coefficient values are low in both these situations due to the stands having been regularly thinned, the texture parameters can be high because of the presence of substantial areas of rock and soil, and the diverse understorey vegetation yielding highly textured images (Fig. 5). This problem might be addressed by combining spectral or shape properties of segments with the texture features to estimate the Gini coefficient more efficiently.

This study has used the available forest survey data measured as part of forest management plan preparation. It can be expected that a stronger relation might be found if a more targeted sampling approach [e.g., 37] was undertaken aimed at measuring the Gini coefficient. We recommend the scale parameter of 40 for mapping the Gini coefficient if the existing sampling plot data is used. However, if a specific sampling strategy apart from the traditional one is undertaken as the basis for determining the Gini coefficient, then smaller segment scales might be more suitable for modeling the Gini coefficient.

The satellite data used in this project was acquired in spring (May). As the spectral differences among plant communities are dependent on the season, estimation of Gini coefficient by means of satellite data might be more reliable if data from other seasons was also used. In addition to the textural parameters belonging to “contrast” group, the measures of “orderliness” and “descriptive statistics” groups can be also investigated in any further studies.

## Figures and Tables

**Table 1.** The Gini and Shannon values calculated from the three example plot data which represent low, moderate and high tree size diversity.

	DBH (cm)	GINI coefficient	Shannon Index			

			Diameter Class Width (cm)			

			1	2	3	4
Low tree size diversity	20;20;21;22;23;24;25;26;26;26;27;28;28 29;30;30;31;31;31;32;32;32;32;33;33;33	0.167	2.367	1.705	1.462	1.293

Moderate tree size diversity	20;20;21;22;23;24;25;27;27;27;29;30;32 32;32;34;35;36;38;38;39;40;40;40;45;45	0.270	2.553	2.304	1.883	1.875

High tree size diversity	09;10;10;18;20;20;28;28;28;37;37;38;45 45;45;53;53;53;60;61;61;61;82;82;83;83	0.496	2.058	1.637	1.928	1.593

## References

[1] Seitz B., Katzel R., Kowarik I., Schulz P.M. (2008). Method for identifying and recording harvest stands of regional provenances of indigenous woody species. Allg. Forst. Jagdztg..

[2] Baskent E.Z., Terzioglu S., Baskaya S. (2008). Developing and implementing multiple-use forest management planning in Turkey. Environ. Manage..

[3] Lahde E., Laiho O., Norokorpi Y., Saksa T. (1999). 1999. Stand structure as the basis of diversity index. Forest Ecol. Manag..

[4] Wikstrom P., Eriksson L.O. (2000). Solving the stand management problem under biodiversity-related considerations. Forest Ecol. Manag..

[5] Anonymous Stand Level Biodiversity-Web Based Training Course.

[6] Spies T.A. (1998). Forest structure: A key to the ecosystem. Northwest Sci..

[7] Lexerod N.L., Eid T. (2006). An evaluation of different diameter diversity indices based on criteria related to forest management planning. Forest Ecol. Manag..

[8] Smith W.R., Farrar R.M., Murphy P.A., Yeiser J.L., Meldahl R.S., Kush J.S. (1992). Crown area and basal area relationships for open-grown southern pines for modelling competition and growth. Can. J. Forest. Res..

[9] Norton D.A., Cochrane C.H., Reay S.D. (2005). Crown-stem dimension relationships in two New Zealand native forests. New Zeal. J. Bot..

[10] Varga P., Chen H.Y.H., Klinka K. (2005). Tree-size diversity between single- and mixed-species stands in three forest types in western Canada. Can. J. Forest. Res..

[11] Rouvinena S., Kuuluvainen T. (2005). Tree diameter distributions in natural and managed old Pinus sylvestris-dominated forests. Forest Ecol. Manag..

[12] Sterba H., Zingg A. (2006). Distance dependent and distance independent description of stand structure. Allg. Forst. Jagdztg..

[13] Chauhan H.B., Nayak S. (2005). Land use/land cover changes near Hazira region, Gujarat using remote sensing satellite data. Photonirvachak-Journal of the Indian Society of Remote Sensing.

[14] Ozdemir I., Koch B., Asan U., Gross C.P., Hemphill S. (2007). Separation of citrus plantations from forest cover using landsat imagery. Allg. Forst. Jagdztg..

[15] Reddy C.S., Pattanaik C., Murthy M.S.R. (2007). Assessment and monitoring of mangroves of Bhitarkanika Wildlife Sanctuary, Orissa, India using remote sensing and GIS. Curr. Sci. India.

[16] Ismail R., Mutanga O., Bob U. (2007). 2007. Forest health and vitality: the detection and monitoring of Pinus patula trees infected by Sirex noctilio using digital multispectral imagery. Southern Hemisphere Forestry Journal.

[17] Kadiogullari A.I., Baskent E.Z. (2008). Spatial and temporal dynamics of land use pattern in Eastern Turkey: a case study in Gumushane. Environ. Monit. Assess..

[18] Keles S., Sivrikaya F., Cakir G., Kose S. (2008). Urbanization and forest cover change in regional directorate of Trabzon forestry from 1975 to 2000 using landsat data. Environ. Monit. Assess..

[19] McCleary A.L., Crews-Meyer K.A., Young K.R. (2008). Refining forest classifications in the western Amazon using an intra-annual multitemporal approach. Int. J. Remote Sens..

[20] St-Onge B., Hu Y., Vega C. (2008). Mapping the height and above-ground biomass of a mixed forest using lidar and stereo Ikonos images. Int. J. Remote Sens..

[21] Gunlu A., Sivrikaya F., Baskent E.Z., Keles S., Cakir G., Kadiogullari A.I. (2008). Estimation of stand type parameters and land cover using Landsat-7 ETM image: A case study from Turkey. Sensors.

[22] Puhr C.B., Donoghue D.N.M. (2000). Remote sensing of upland conifer plantations using Landsat TM data: a case study from Galloway, south-west Scotland. Int. J. Remote Sens..

[23] Innes JL., Koch B. (1998). Forest biodiversity and its assessment by remote sensing. Global Ecol. Biogeogr..

[24] Aynekulu E., Kassawmar T., Tamene L. (2008). Applicability of ASTER imagery in mapping land use/cover as a basis for biodiversity studies in drylands of northern Ethiopia. Afr. J. Ecol..

[25] Bawa K., Rose J., Ganeshaiah K.N., Barve N., Kiran M.C., Umashaanker R. (2002). Assessing Biodiversity from Space: an Example from the Western Ghats, India. Conserv. Ecol..

[26] Levin N., Shmida A., Levanoni O., Tamari H., Kark S. (2007). Predicting mountain plant richness and rarity from space using satellite-derived vegetation indices. Divers Distrib..

[27] Cho V.H.K. (2004). Investigations for segment-based classification of satellite images for the purpose of forest mapping. Allg. Forst. Jagdztg..

[28] Ozdemir I., Asan U., Koch B., Yesil A., Ozkan U.Y., Hemphill S. (2005). Comparison of Quickbird-2 and Landsat-7 ETM+ data for mapping of vegetation cover in Fethiye-Kumluova coastal dune in the Mediterranean region of Turkey. Fresen. Environ. Bull..

[29] Mallinis G., Koutsias N., Tsakiri-Strati M., Karteris M. (2008). Object-based classification using Quickbird imagery for delineating forest vegetation polygons in a Mediterranean test site. Isprs J. Photogramm..

[30] Ozdemir I. (2008). Estimating stem volume by tree crown area and tree shadow area extracted from pansharpened Quickbird imagery in open Crimean juniper forests. Int. J. Remote Sens..

[31] Definiens A.G. (2006). Definiens Professional 5 Reference Book.

[32] Baatz M., Benz U., Dehgani S., Heynan M., Holtje A., Hofmann P., Lingenfelder I., Mimler M., Sohlbach M., Weber M., Willhauck G. (2001). eCognition Object Oriented Image Analysis User Guide.

[33] Tian J., Chen D.M. (2007). Optimization in multi-scale segmentation of high-resolution satellite images for artificial feature recognition. Int. J. Remote Sens..

[34] Shannon C.E., Shannon C.E., Weaver W. (1948). The mathematical theory of communication. In Mathematical Theory of Communication.

[35] Staudhammer C.L., LeMay V.M. (2001). Introduction and evaluation of possible indices of stand structural diversity. Can. J. Forest. Res..

[36] Hall-Beyer M. (2007). GLCM tutorial home page. http://www.fp.ucalgary/ca/mhallbey/tutorial.htm.

[37] Staupendahl K. (2008). The modified six-tree-sample - a suitable method for forest stand assessment. Allg. Forst. Jagdztg..

